# ﻿Complementary description of *Peltidiumnayarit* (Copepoda, Harpaticoida, Peltidiidae) and a new record at Revillagigedo Archipelago, Mexico

**DOI:** 10.3897/zookeys.1214.132950

**Published:** 2024-10-04

**Authors:** José Ricardo Palomares-García, Jaime Gómez-Gutiérrez

**Affiliations:** 1 Departamento de Plancton y Ecología Marina, Centro Interdisciplinario de Ciencias Marinas, Instituto Politécnico Nacional, C.P. 23096, La Paz, BCS, Mexico Centro Interdisciplinario de Ciencias Marinas, Instituto Politécnico Nacional La Paz Mexico

**Keywords:** Crustacean fauna, Harpaticoida, marine copepods, range extension, taxonomy

## Abstract

Four adult females of a rare benthic/epiphytic harpacticoid copepod species of the genus *Peltidium* Philippi, 1839 were collected from insular zooplankton samples obtained at Revillagigedo National Park, Mexico. They were identified as *Peltidiumnayarit* Suárez-Morales & Jarquín-González, 2013 with type location (and the single previously known distribution site) at Playa Careyeros (20°46'59.46"N, 105°30'35.48"W), Nayarit, Mexico. We provide a complementary description of this species including new details of antennules, caudal rami, legs 1 and 5, and cuticular ornamentation using scanning electron microscopy observations. *Peltidiumnayarit* has a dark reddish-pink coloration allegedly mimicking the color of the macroalgae where they live, but specimens collected in the present study were obtained from sea surface zooplankton net tows. The record of *Peltidiumnayarit* in the Revillagigedo Islands represents the southwestern-most record of the genus in the Americas and the second record for Mexico.

## ﻿Introduction

The copepod genus *Peltidium* currently includes 47 nominal species widely distributed in different regions of the world’s oceans (Walter and Boxshall 2024). However, *Peltidium* species are typically found in low species richness in each region (Suárez-Morales and Jarquín-González 2013). Five *Peltidium* species have been reported in the Caribbean region (Varela 2005; Suárez-Morales et al. 2006; Varela and Gómez 2013). Only two species of the subfamily Peltidiinae have been reported in the Eastern Tropical Pacific (Gómez and Varela 2013; Suárez-Morales and Jarquín-González 2013). Gómez and Varela (2013) described a new Peltidiidae species, *Alteuthaalsagopu* Gómez & Varela, 2013, collected on the coast of Sinaloa, Gulf of California, Mexico. *Peltidiumnayarit* Suárez-Morales & Jarquín-González, 2013, is therefore the second record of Peltidiidae known in the Mexican Pacific. *Peltidiumnayarit* was discovered in meiobenthic samples collected from a rocky, sandy beach in the state of Nayarit, Mexico. The original description of *P.nayarit* was based only on optical microscopy. The goal of the present study was to complement the original morphological description with the description of females observed with scanning electron microscopy and report the offshore distribution range of this species previously known only from the neritic region of the Central Mexican Pacific.

## ﻿Materials and methods

We carried out a pioneering biological survey of the insular zooplankton diversity of the four islands of the Revillagigedo National Park (San Benedicto, Roca Partida, Clarion, Socorro), Mexico, from 18–22 April 2023 on board the *L/A Quino El Guardian*. Quantitative zooplankton samples were collected with a standard zooplankton net (0.6 m mouth diameter, 2 m length, 300 µm mesh size), towed near the sea surface (<2 m depth) for 10 minutes. The net was equipped with a General Oceanic digital flowmeter (GO R2030) to estimate the filtered seawater volume and calculate standardized abundance expressed in ind./1000 m^3^ (Smith and Richardson 1977). Copepods were fixed in a 4% formalin solution buffered with saturated sodium borate, then sorted from the original samples and transferred to 70% ethanol with glycerin for long-term preservation. All harpacticoid copepods were sorted out from the entire zooplankton samples. Two harpacticoid copepod specimens of a *Peltidium* species were observed with a scanning electron microscope (SEM, Hitachi S-3000N) in the Laboratory of Scanning Electron Microscopy located at Centro de Investigaciones Biológicas del Noroeste S.C., La Paz, Baja California Sur, Mexico. This methodology stands out due to its high resolution and great depth of field, which allows a three-dimensional visualization of the copepods. Each specimen was gradually dehydrated for one hour at each of the following ethanol concentrations: 70, 80 and 96%. The harpacticoid copepods were later dried to the critical point with carbon dioxide gas (CO_2_) using the Polaron E3000 critical point equipment (Samdri PVT 3B). The copepods were mounted on a copper filament with sticky glue to observe the specimens from different angles in the SEM. The specimens were coated with gold ions using a Polaron E5100 sputter-coater (Denton Vacuum Desk II). The observations were made with an intensity of 20 kV capable of a maximum of 300,000 × magnification. The other two *Peltidium* harpacticoid specimens were observed using an optical stereo microscope (Carl Zeiss Stemi 2000).

Digital images of the *Peltidium* specimens were obtained in dorsal, ventral, lateral and frontal views. Taxonomic diagnostic characters of this species were described in detail from anterior to posterior appendages. The images were improved in contrast and brightness and transparency of the background using the digital analysis of layers with Photoshop CS5 (v.12.0 x64). Plates of each specimen were made with a black background to contrast the SEM images using Adobe Illustrator CS5 (v. 15.0). The copepod specimens examined in the present study (including both specimens observed with SEM) were deposited in the Zooplankton Collection, Centro Interdisciplinario de Ciencias Marinas, Instituto Politécnico Nacional, La Paz, Baja California Sur, Mexico (Curator José Ricardo Palomares-García).

## ﻿Description

### ﻿ Peltidium nayarit

Taxon classificationAnimaliaHarpacticoidaPeltidiidae

Suárez-Morales & Jarquín-González, 2013

3B1AE73E-2792-5A66-B321-387955B54DF4

Figs 1 , 2 , 3

#### Material examined.

• Four adult females, two specimens mounted on glycerin sealed with acrylic varnish for optical stereo microscope observation and two specimens used for scanning electron microscopy • Two adult females collected at Punta Norte, San Benedicto Island, Revillagigedo National Park, Mexico (19°20'10"N, 110°47'58"W); 18 April 2023, 16:10 h, zooplankton sample collected near surface < 2 m depth; collector Jaime Gómez-Gutierrez • One adult female collected at Punta Sureste, Clarion Island, Revillagigedo National Park, Mexico (18°20'50"N, 114°41'39"W); 21 April 2023, 18:47 h, zooplankton sample collected near surface < 2 m depth; collector Jaime Gómez-Gutierrez • One adult female collected at Piedra Caleta, Clarion Island, Revillagigedo National Park, Mexico (18°22'17"N, 114°41'35"W); 23 April 2023, 18:47 h, zooplankton sample collected near surface < 2 m depth; collector Jaime Gómez-Gutierrez. Standardized abundance of *P.nayarit* was typically low (average 9.7 ind./1000 m^3^, range 7.2–12.5 ind./1000 m^3^), found in three positive sampling stations out of a total of 10 zooplankton sampling stations.

#### New locality.

San Benedicto and Clarion islands, Revillagigedo National Park, Mexico.

#### Complementary description.

We describe only the observed morphological differences and details not included in the original description of *Peltidiumnayarit* reported in Suárez-Morales and Jarquín-González (2013).

**Figure 1.**
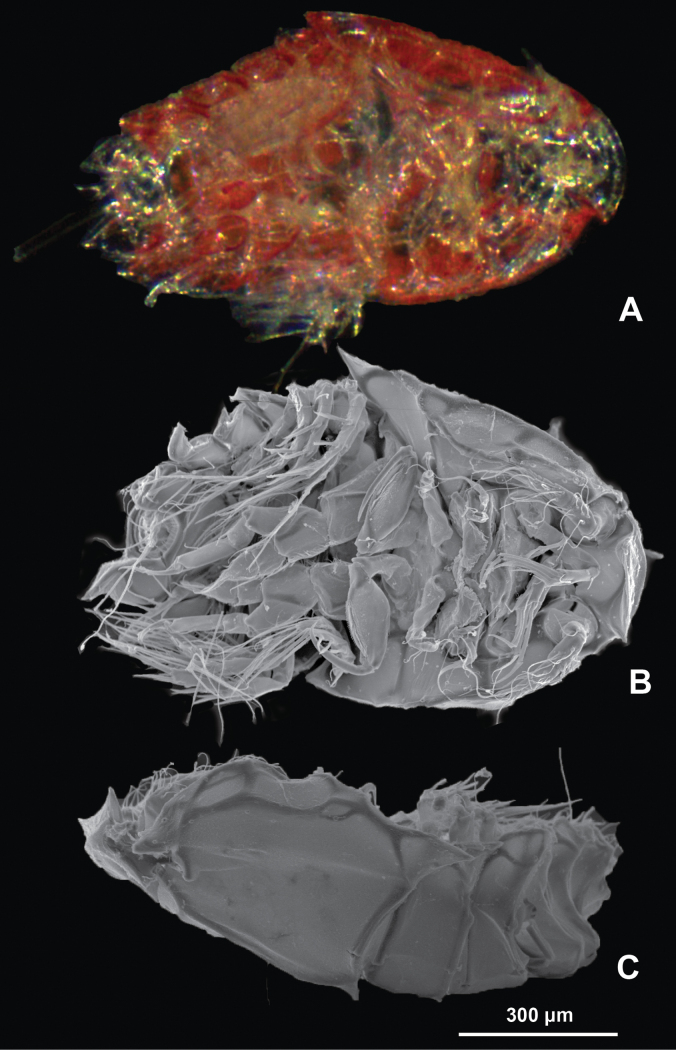
*Peltidiumnayarit* adult females collected from San Benedicto and Clarion Islands, Revillagigedo National Park, Mexico **A** photograph of a female showing dark reddish-pink coloration in ventral view **B** SEM image of a specimen in ventral view **C** SEM image of a specimen in right lateral view.

**Figure 2.**
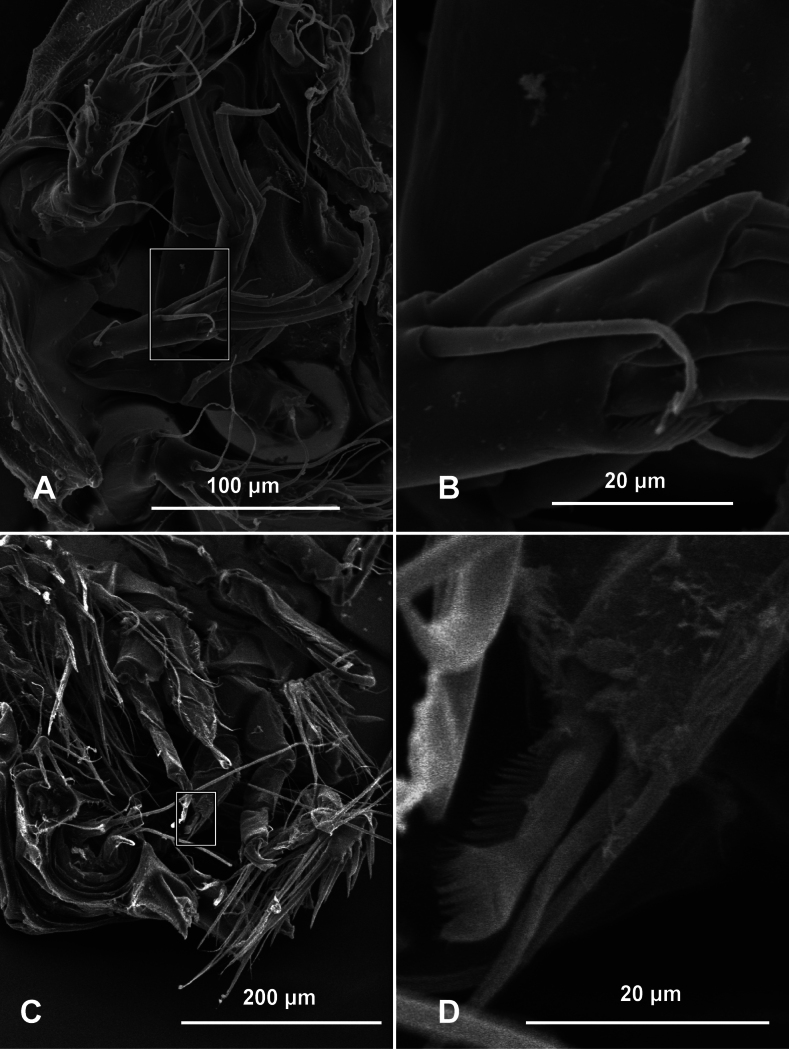
*Peltidiumnayarit* adult female collected from San Benedicto and Clarion Islands, Revillagigedo National Park, Mexico **A** antenna with ornamented inner pectinate margin **B** detail of the pectinate margin **C** leg 1 with a pectinate fringe **D** detail of the inner margin of the second endopodite segment.

Copepods with dark reddish-pink coloration (Fig. 1A). Specimens measure on average 0.8 mm total length and 0.5 mm width (Fig. 1B, C). Free endopodal segment with outer row of long spinules, armed with one spine and two lateral setae plus seven distal setal elements, four of them being articulated stout setae. The same element is ornamented with inner pectinate margin (Fig. 2A, B). ***Antenna*.** Coxa small, allobasis with short abexopodal seta and longitudinal patch of spinules on outer margin. Exopod two-segmented, elongated, first segment with short slender seta, second segment bearing three setae distally, distal margin with row of short spinules. One exopodal seta modified, with regular pectinate ornamentation along both margins (Suárez-Morales and Jarquín-González 2013, Fig. 2C). The pectinate ornamentation of the distal margin of the antenna was not shown in the original description of *P.nayarit* (Suárez-Morales and Jarquín-González 2013).

***Leg 1*** (Fig. 2D). Coxa elongate, ornamented with single row of small spinules on inner and outer margins, plus single short seta on inner middle part of segment. Basis wide, inner margin and part of outer margin ornamented with spinules. Inner distal basipodal seta reaching distal margin of first endopodal segment. Outer basipodal seta reaching distal end of basis. ***Exopod*** three-segmented, second exopodal segment longest, about 1.5 times as long as first segment, with patch of minute spinules on outer distal margin. Two exopodal claws on distal position of third exopodal segment; outer claw half as long as inner claw. ***Endopod*** two-segmented, shorter than exopod. Endopodal segments wide, globose (sensu Wells 2007), ornamented with rows of short setule along the inner and outer margins. Terminal elements include a spine ornamented with distal row of minute spinules and two equally long slender setae. First endopodite segment with an inner seta; second endopodite segment with an inner seta and two equally long slender terminal setae, the inner seta bears a pectinate fringe on its inner margin (Fig. 2C, D).

#### Remarks.

In the original description of *P.nayarit* (Suárez-Morales and Jarquín-González 2013, fig. 2C) the margin of the endopodite with the pectinate fringe on its inner margin was not described (Fig. 2C, D).

**Figure 3.**
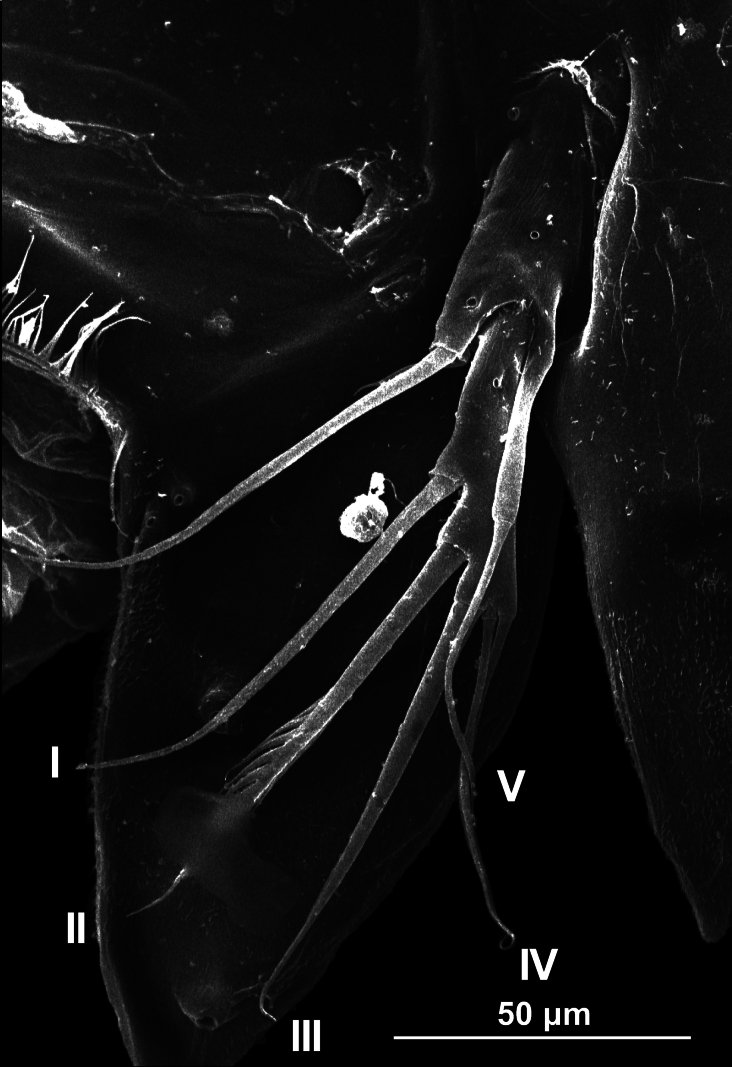
Leg 5 of *Peltidiumnayarit*, adult female, showing exopodal setae. Setal nomenclature follows Wells (2007) and Suárez-Morales and Jarquín-González (2013). Specimen collected from San Benedicto Island, Revillagigedo National Park, Mexico.

Leg 5 (Fig. 3). This is the amendment of the original description exopod and baseoendopod separated. Baseoendopod with two pores bearing single inner seta; external seta long, borne on elongate cylindrical lobe of baseoendopod reaching half the length of exopodal lobe; exopodite slender, with one pore and with 5 setal elements (I–V) (sensu Wells 2007), two inserted on inner margin (I, II), and three (III–V) distally; elements I–III represented by stout, distally pinnate setae, elements IV and V possess equally long slender setae; in the specimens from the Revillagigedo Islands, the unique pinnate element observed is element III, and elements I and II were spiniform (Fig. 2E).

According to Suárez-Morales and Jarquín-González (2013), *P.nayarit* shares some diagnostic morphological characters with *P.speciosum* Thompson & Scott, 1903, including leg 5 with a very similar armature and structure except for a relatively robust exopodal segment (length/width ratio = 3.7 vs. 4.2 in *P.nayarit*) and a shorter outer baseoendopodal seta, reaching to about half the length of exopodal seta V (Nicholls 1941, see fig. 8). These morphological features differ from *P.nayarit*, in which the same seta almost reaches the distal end of seta V (Suárez-Morales and Jarquín-González 2013, fig. 3). However, we observed remarkable morphological differences with the original description, because the III element of the exopodite of the specimens of the present study bear only pinnate element and elements I and II were spiniform, while in specimens of *P.nayarit* from the Nayarit coast elements I–III have setae.

## ﻿Discussion

Several harpacticoid genera have been discovered and described as meiobenthic fauna. The meiobenthic habitat is commonly exposed to strong seawater currents, where the low-profile body shape (like an isopod) helps the copepod maintain its position on the substrate’s surface or remain attached to coral or macroalgae (Noodt 1971; Hicks 1980; Gheerardyn et al. 2006). Overall, sampling effort in phytal-meiobenthic habitats has been scarce and has received little attention in most regions of the world. The original discovery and single record of *Peltidiumnayarit* was from a rocky beach (20°46'59.46"N, 105°30'35.48"W, Nayarit, central Pacific of Mexico) as a result of resuspension of sediments by turbulence from the meiobenthic community into the water (Suárez-Morales and Jarquín-González 2013).

We report the second record of this species from two distant oceanic islands at Revillagigedo National Park, San Benedicto and Clarion Islands. The San Benedicto record represents an unexpected distant habitat about 600 km offshore from the type locality on the Nayarit coast; Clarion Island is a further 425 km offshore from San Benedicto Island. This broad distribution range may be related to the influence of episodic storms and hurricanes causing anomalous river plumes that favor improbable zooplankton transport between the mainland (Nayarit and Colima states) and the Islas Marias Archipelago, four islands located close to the shelf-break off the central Mexican Pacific coast (Martínez-Flores et al. 2011; Gómez-Gutiérrez et al. 2014). However, other oceanographic processes, such as eddies from Cabo San Lucas and Cabo Corrientes with a typical southwest offshore trajectory (Kurczyn et al. 2012) can cause the drift of offshore zooplankton to reach the insular habitats of Revillagigedo Archipelago. *Peltidiumnayarit* in its planktonic phase is allegedly a low-abundance copepod species that likely attains larger population densities in the epibenthic habitat, which will be investigated in future studies.

## Supplementary Material

XML Treatment for Peltidium nayarit

## ﻿Additional information

### Author ORCIDs

José Ricardo Palomares-García https://orcid.org/0000-0002-0054-9656

Jaime Gómez-Gutiérrez https://orcid.org/0000-0003-2516-897X

### Data availability

All of the data that support the findings of this study are available in the main text.
